# Early career member highlights from the 22nd ERS Lung Science Conference: development of chronic lung diseases – from life-spanning mechanisms to preventive therapy

**DOI:** 10.1183/23120541.00659-2024

**Published:** 2024-12-16

**Authors:** Cheng-Yu Wu, María Camila Melo-Narváez, Sara Cuevas-Ocaña

**Affiliations:** 1 Excellence Cluster Cardio-Pulmonary Institute, Universities of Giessen and Marburg Lung Center, Member of the German Center for Lung Research (DZL), Justus Liebig University, Giessen, Germany; 2 Institute for Lung Research, Philipps-University Marburg, Member of the DZL, Marburg, Germany; 3 Comprehensive Pneumology Center, Institute of Lung Health and Immunity, Helmholtz Center Munich, Member of the DZL, Munich, Germany; 4 Biodiscovery Institute, Translational Medical Sciences, School of Medicine, University of Nottingham, Nottingham, UK; 5 These authors contributed equally

## Abstract

The 22nd Lung Science Conference (LSC) organised by the European Respiratory Society (ERS) took place on the 14–17 March 2024 online and in person in Estoril, Portugal. During these less than four days, basic and clinical researchers and other experts with an interest in translational science [1] got together to share the best international lung research presented in a common programme, and enjoyed fruitful debates and interactions in numerous social opportunities for networking. Early career scientists and established investigators had the opportunity to get deeply immersed in new discoveries around the origin, development and potential treatments of numerous respiratory diseases such as COPD and lung fibrosis.

## Shareable abstract

**ERJOR: In case you missed the #LSC2024, this work highlights some of the new key points that were discussed at the @EuroRespSoc Lung Science Conference about #COPD & #IPF by @SaraOcana1 @danielchengyuwu @cami93melo @EarlyCareerERS @ERSpublications #ERJOR** https://bit.ly/4cbMjpS

The 22nd Lung Science Conference (LSC) organised by the European Respiratory Society (ERS) took place on the 14–17 March 2024 online and in person in Estoril, Portugal. During these less than four days, basic and clinical researchers and other experts with an interest in translational science [1] got together to share the best international lung research presented in a common programme, and enjoyed fruitful debates and interactions in numerous social opportunities for networking. Early career scientists and established investigators had the opportunity to get deeply immersed in new discoveries around the origin, development and potential treatments of numerous respiratory diseases such as COPD and lung fibrosis. ERS early career members have summarised here a few key new messages about COPD and idiopathic pulmonary fibrosis (IPF) that were presented at the LSC 2024, but the full programme can be accessed in the ERS Respiratory Channel (https://channel.ersnet.org/hp-60-1-home-replay).

Unlike previously thought, novel findings presented at the LSC 2024 suggest that COPD is not solely a disease of ageing but a complex disease with multiple risk factors and pathological features that develop throughout the whole lifespan (figure 1). Particularly, a new paradigm, termed GETomics, has been proposed at the LSC2024 illustrating that pathogenesis of chronic lung diseases such as COPD is driven by cumulative gene (G)–environment (E) interactions encountered during individuals’ lifes (time, T) [2]. From the moment of conception to early adulthood, several factors can contribute to impaired lung development trajectory. For example, genome-wide association studies have revealed COPD-associated genetic risk loci, including variants in the *Serpin family A member 1/ α_1_-antitrypsin* (*SERPINA1*), *Family with sequence similarity 13 member A* (*FAM13A*) and *Hedgehog interacting protein* (*HHIP*) presented at the LSC 2024 [3].

**FIGURE 1:**
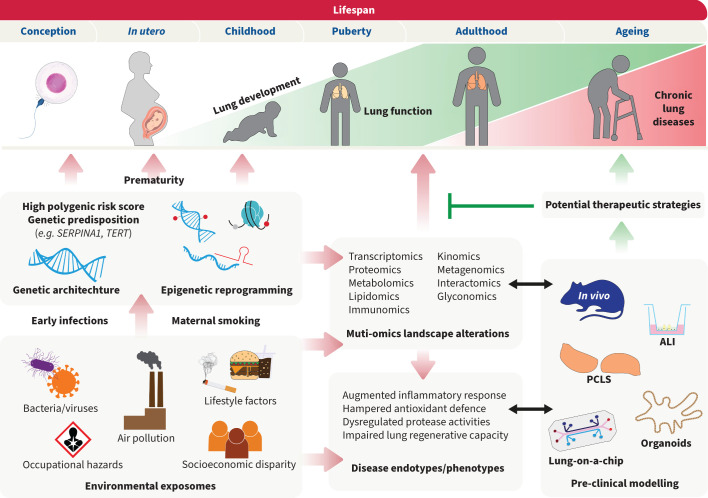
Life-spanning mechanisms underlying the development of chronic lung diseases. The complex interplay of genetic, epigenetic and environmental factors contributes to lung health throughout an individual's lifetime. Red arrows indicate the events encountered in a lifespan that contribute to chronic lung diseases development. Genetic predispositions and early-life events can lead to impaired lung development. During adulthood, environmental exposures, lifestyle choices and socioeconomic circumstances can further exert negative effects on lung function. These elements intertwine throughout our lives, shaping the risk of chronic lung ailments as we age. Early detection and development of novel therapeutical interventions are essential to prevent chronic lung diseases, as indicated by green arrows. Pre-clinical models, including *in vivo* studies, air–liquid interface (ALI) culture, precision-cut lung slices (PCLS), organoids and lung-on-a-chip, can aid in elucidating molecular alterations associated with lung disease development. As such, insights gained from these models can help identify biomarkers for early diagnosis and develop therapies tailored to susceptible populations, ultimately improving lung health outcomes across the lifespan.

During the perinatal period, exposure to noxious agents, such as cigarette smoke, has lasting impacts on lung function [4]. Conversely, lower exposure to airborne pollutants, including particulate matter with diameter ≤2.5 µm, black carbon and nitrogen oxides, is associated with better lung function development from childhood to young adulthood [5]. Maternal diet, such as intake of antioxidant-rich fruits or polyunsaturated fatty acid-high foods, can mitigate the harmful effects of environmental exposure and may influence offsprings’ body mass index trajectories from birth to the early childhood, which is associated with children's lung function [6, 7] as shown at the LSC 2024. These findings align with the developmental origins of health and disease (DOHaD) concept, highlighting how early-life events have lasting effects on health outcomes.

Environmental exposomes in adulthood greatly contribute to the decline rate of the lung function. These factors include all exogenous stressors an individual encounters throughout life including, but not limited to, cigarette smoke, electronic cigarette (e-cigarette) vapour, infections and lifestyle factors [8, 9]. Chronic exposure to harmful agents can lead to long-lasting effects *via* altering the epigenetic landscape [10], affecting gene expression patterns and increasing susceptibility to COPD development and progression [11, 12]. Investigating mechanisms of epigenetic regulation may provide novel avenues for biomarker identification and targeted therapies [10].

To unravel mechanisms underlying COPD development across a lifespan, scientists have established pre-clinical models aiming to bring new avenues to the COPD field. Animals with *HHIP* haploinsufficiency develop age-related emphysema without environmental triggers, recapitulating human genetic predisposition to COPD development [3]. Moreover, impaired fibroblast growth factor (FGF)-10 signalling, essential for lung development, accelerates emphysema development in mice upon cigarette smoke exposure [13]. Mouse *in utero* smoking, to model the impact of external stressors during perinatal periods, increases epigenetic signatures through *cytochrome P450 family 1 subfamily A member 1* (*Cyp1a1*) DNA methylation in offspring. In line with the DOHaD concept, this effect lasts through early adulthood and can be re-established in the later life of the offspring upon cigarette smoke exposure [4]. Similarly, prenatal adversities influence immune response to viral infections, which was shown at the LSC 2024 to be gender-specific [14]. Exogenous stress from substances in e-cigarette aerosols alters adult mouse lung structures [9].

In addition to *in vivo* models, an *in vitro* model reveals carcinoembryonic antigen cell adhesion molecule 6-dependent coping mechanisms against nitrosative/oxidative stress, potentially expanding the COPD biomarker panel to identify susceptible smokers [15]. As shown in an *ex vivo* model of precision-cut lung slices (PCLSs), cigarette smoke can hamper immunoproteasome machinery and may increase susceptibility to viral exacerbation in patients with advanced COPD [16]. Notably, preliminary data presented at the LSC 2024 suggest that reprogramming cells as human-induced pluripotent stem cells (hiPSC) holds immense potential to develop new pre-clinical models for COPD, such as hiPSC-derived airway epithelium culture on an air–liquid interface (ALI) or human lung organoids [17].

Most importantly, several potential therapeutic strategies for COPD have been identified using the preclinical models. Combining lymphotoxin β-receptor blockers and Wnt signalling activators promotes lung regeneration in mice with well-established emphysema [18]. Supplementing FGF-10 reverses emphysema and the remodelled pulmonary vasculature in a mouse model of cigarette smoke- and elastase-induced injuries [13]. In addition to the published findings, the latest advances in COPD therapeutic strategies were unveiled at the LSC 2024. For example, reducing oxidative stress *via* an inhibitor of p70-S6K to induce sirtuin 1 levels can reverse senescence phenotype in lung epithelial cells [19]. Moreover, lipid nanoparticle (LNP)-encapsulated small interfering (si)RNA-targeted SAM-pointed domain-containing transcription factor knockdown on ALI culture highlights a potential LNP-siRNA treatment for COPD patients [20]. Finally, therapies using hiPSC-derived cells to target epigenetic modifications may facilitate lung repair in a range of pulmonary diseases beyond COPD [17].

IPF is a devastating chronic lung disease with a survival median of 3–5 years after diagnosis [21]. Although IPF has an unknown aetiology, the study of familial IPF and early onset cases led to the identification of specific genetic variants associated with familial cases of IPF. In this case, animal models and *in vitro* models with specific genetic traits have been generated using genetic engineering [22–24], as presented at the LSC 2024, to help understand the role of genetics in lung diseases. For example, genetic mutations in regions codifying for proteins responsible for telomere maintenance (*Telomerase reverse transcriptase*, *TERT*), mucociliary clearance (*Mucin 5B*, *MUC5B*) [25] or alveolar maintenance (*Surfactant protein C*, *SFTPC*) were shown at the LSC 2024 to induce spontaneous fibrosis in mice [26].

However, more evidence suggests that the interaction between our genes (G) and our environment (E) during our lives (T) is what defines the response of our lungs to certain insults, which could lead to a higher susceptibility to chronic lung diseases later in life [2] (figure 1). Indeed, recent population-based screening studies presented at the LSC 2024 suggest that having a reduced lung function at the age of 25 years old, associated with prematurity [27] or respiratory infections in the childhood [2], can lead to a higher susceptibility to chronic lung diseases such as IPF during adulthood. This susceptibility can be further exacerbated when combined with cigarette smoking, alcohol consumption or work-related environmental exposures [2].

Considering that gene–environment–time (GET) interactions increase with age [2], it is not a surprise that the incidence of chronic lung diseases such as IPF dramatically increases after 65 years of age. Accordingly, several hallmarks of ageing, such as cellular senescence, mitochondrial dysfunction [28] and epigenetic alterations [10], have been found to be increased in IPF. Transcriptomics and spatial-based studies have revealed that all these molecular alterations can lead to impaired crosstalk among different cell types in the aged lung. For example, both the alveolar epithelium and the mesenchyme, usually responsible for alveolar regeneration, undergo epigenetic changes that reduce their differentiation capacity in response to injury. This leads to accumulation of intermediate profibrotic states such as aberrant basaloid cells [28] and secreted frizzled-related protein 1-positive/secreted phosphoprotein 1-positive inflammatory fibroblasts [26] in the aged lungs that perpetuate inflammation and extracellular matrix deposition, and impair the regeneration of the alveolar epithelium. New efforts in integrating these omics-based studies with clinical imaging such as micro-computed tomography presented at the LSC 2024 have shown promising results in identifying the origin of these altered cellular states in early disease stages [29], which in the future, could be used to improve our diagnostic options for lung fibrosis.

At the LSC 2024, several human *in vitro* models, such as organ-on-chip [30], PCLSs [31], or organoids [26, 32], that recapitulate early ageing-associated fibrotic processes were highlighted as unique platforms for the discovery of novel drugs to target the disease at a more effective point. In this direction, studies based on PCLSs provided insights into the efficacy of potential new therapies for IPF such as the antifibrotic imatinib [33] or senolytics such as dasatinib/quercentin [31].

In summary, an individual can experience multiple “hits” or harmful exposures throughout their lifetime that cumulatively contribute to chronic lung diseases, such as COPD and IPF. Collaborative efforts between basic and clinical scientists are essential – by using advanced disease models, and integrating genetic, environmental and clinical data – to decipher gene–environment interactions over time [2], which will help implement diagnostic and interventional strategies in the clinic as early as possible in order to increase our capacity to predict disease onset at earlier time points and thereby reduce the incidence of chronic lung diseases late in life. Events such as the LSC provide ideal platforms to facilitate the interactions among respiratory experts with different and complementary views and expertise that would speed up the progress of respiratory medicine.
